# Cannibalism in invasive, native and biocontrol populations of the harlequin ladybird

**DOI:** 10.1186/1471-2148-14-15

**Published:** 2014-02-05

**Authors:** Ashraf Tayeh, Arnaud Estoup, Eric Lombaert, Thomas Guillemaud, Natalia Kirichenko, Lori Lawson-Handley, Patrick De Clercq, Benoît Facon

**Affiliations:** 1 INRA, UMR, 1062, CBGP Montpellier, France; 2 INRA, UMR 1355 Institut Sophia Agrobiotech, F-06903 Sophia Antipolis, France; 3 Université de Nice-Sophia Antipolis, UMR Institut Sophia Agrobiotech, F-06903 Sophia Antipolis, France; 4 CNRS, UMR 7254 Institut Sophia Agrobiotech, F-06903 Sophia Antipolis, France; 5 V.N. Sukachev Institute of Forest, Siberian Branch of Russian Academy of Sciences, Akademgorodok 50/28, Krasnoyarsk 660036, Russia; 6 Evolutionary Biology Group, School of Biological, Biomedical and Environmental Sciences, University of Hull, Cottingham Road, Kingston-Upon-Hull HU6 7RX UK; 7 Department of Crop Protection, Ghent University, Coupure Links 653, B-9000 Ghent, Belgium

**Keywords:** *Harmonia axyridis*, Cannibalism, Evolution, Invasive, Native and biocontrol populations

## Abstract

**Background:**

Cannibalism is widespread in both vertebrates and invertebrates but its extent is variable between and within species. Cannibalism depends on population density and nutritional conditions, and could be beneficial during colonisation of new environments. Empirical studies are needed to determine whether this trait might facilitate invasion of a new area in natural systems. We investigated whether the propensity for cannibalism in *H. axyridis* differs both between native and invasive populations and between invasive populations from the core and from the front of the invasive area in Western Europe. We also compared the propensity for cannibalism of these natural populations with that of laboratory-reared biocontrol populations. We measured the cannibalism rates of eggs by first instar larvae and adult females at two different individual densities of ladybirds from three types of population (invasive, native and biocontrol), in laboratory-controlled conditions.

**Results:**

Cannibalism was significantly greater in larvae from invasive populations compared to native or biocontrol populations, but there was no difference in cannibalism rates between populations from the core or front of the invaded range. Cannibalism was significantly lower in larvae from biocontrol populations compared to wild (invasive and native) populations. No differences in cannibalism rates of adult females were found between any populations. While high population density significantly increased cannibalism in both larvae and adults, the norm of reaction of cannibalism to individual density did not change significantly during the invasion and/or laboratory rearing processes.

**Conclusion:**

This study is the first to provide evidence for a higher propensity for cannibalism in invasive populations compared to native ones. Our experiments also shed light on the difference in cannibalism evolution with respect to life stages. However, we are still at an early stage in understanding the underlying mechanisms and several different research perspectives are needed to determine whether the higher propensity for cannibalism is a general feature of the invasion process.

## Background

Cannibalism is ubiquitous in the animal kingdom [1,2] and probably occurs in almost all major vertebrate and invertebrate groups [3]. It is particularly common in arthropods [4,5], notably in generalist predators, where it is seen as an extension of their normal predatory behaviour [6]. One common feature of cannibalism in arthropods is that victims are preferentially defenceless life stages such as eggs, young larvae or quiescent pupae [2,7]. The extent of cannibalism is highly variable between, but also within species [8]. Significant genetic variation in cannibalism rate has been shown within several species (see for instance [1,9-11]. Moreover, experimental selection studies on the red flour beetle *Tribolium castaneum* have revealed that it is possible to select rapidly for strains displaying different levels of cannibalism [12,13].

Various factors can be responsible for the evolution of cannibalism rate. On the one hand, cannibalism may provide two clear benefits. First, conspecifics can be a high-quality resource, thus cannibalism may be adaptive due to its nutritional benefit, particularly when resources are limited [2,14,15]. Second, it may decrease the intensity of competition [3,16]. On the other hand, several costs may select against cannibalism, since cannibals risk acquiring pathogens or being injured or killed [3,17]. Also, the evolution of cannibalism in structured populations may be limited by kin selection, since individuals risk an inclusive fitness cost by eating related individuals [18,19]. In relation to this, a theoretical study has recently examined how different levels of dispersal affect the evolution of cannibalism and how cannibalism in turn drives the evolution of dispersal [8]. This study showed that the coevolution of cannibalism and dispersal results in the evolution of various alternative life-history strategies, with different dispersal and cannibalism regimes depending on the environmental conditions that determine initial cannibalism rates. For instance, higher dispersal rates increase the propensity for cannibalism by decreasing the relatedness between the cannibals and their prey, but high initial cannibalism rate can also drive the evolution of dispersal [8].

Cannibalism has also been suggested to be helpful in colonizing new environments [20,21]. It has been shown experimentally in the red flour beetle that cannibalism might facilitate colonization of a marginal environment by rescuing individuals from nutritionally poor situations through increased survival and fecundity and decreased development time [11]. Moreover, higher cannibalism rates have been shown to evolve in populations colonizing nutritionally stressful environments [11,22]. Via et al. [11] also stressed the need for more empirical work to test whether cannibalism might facilitate invasion of a new area in natural systems. To our knowledge, this question has not yet been investigated. The aim of the present study is to fill this gap by testing differences in cannibalism among various native, invasive and biocontrol populations of the harlequin ladybird *Harmonia axyridis* (Pallas, 1773) (Coleoptera: Coccinellidae).

Native to Asia, *H. axyridis* was introduced to North America and Europe as a biological control agent against aphids, and later turned invasive. Its rapid worldwide spread followed a complex invasion history involving admixture events and a prominent role of the Eastern North American outbreak [23,24]. In Europe, there is evidence for admixture between Eastern North American founders and individuals from the European biocontrol laboratory population, used locally to control aphid populations (with a contribution of biocontrol stock genes estimated around 40%) [23]. *H. axyridis* is an appropriate model for studying cannibalism in the particular context of biological invasion, as well as in that of laboratory-reared biocontrol populations. Firstly, cannibalism appears to play an important role in the population dynamics of *H. axyridis*[25]. In its native area, field studies indicate that egg cannibalism may be a major mortality factor regulating population densities [26], and account for up to 60% of egg mortality [25,27]. Although egg cannibalism is most often performed by larvae, adults can also eat conspecific eggs [7,28,29]. Cannibalism in *H. axyridis* has been shown to provide nutritional benefits when other prey are scarce [20], nutrients are deficient, or toxic [30], and to increase survival and reduce development time [20,30]. Significant genetic variation in cannibalism rate has been demonstrated between individuals within population [20]. Notably, there is a strong genetic basis for the expression of cannibalism in a low food environment, whereas heritability is not significant in a higher food environment [20]. *H. axyridis* displays cannibalism behaviour on both sibling and non-sibling eggs. In aphidophagous coccinellids, including *H. axyridis,* sibling egg cannibalism is frequently associated with the presence of bacteria such as *Wolbachia* and *Spiroplasma*, which kill males early in their development [31,32]. Male-killer infected females obtain a substantial indirect fitness benefit from consuming the undeveloped male eggs from within their clutch [33]. Here, we chose to focus on non-sibling egg cannibalism to avoid any confounding effects of male-killer infection. Moreover, larvae are less likely to cannibalize related than non-related eggs [34]. Finally, we found, using the same two population transects used in this study, a marked heritable increase in flight speed of *H. axyridis* adults from the core to the front of the invasion range in Western Europe (Lombaert E, Estoup A, Joubard B, Facon B, Grégoire JC, Jannin A, Blin A, Thomas G: Rapid evolution of dispersal abilities during the expansion of the invasive ladybird *Harmonia axyridis* in Europe, submitted). As a theoretical study has recently showed, we could expect that this increase of dispersal may have affected the evolution of cannibalism during the spatial expansion.

The main goal of this study was to investigate whether the propensity for cannibalism in *H. axyridis* differs both between native and invasive populations and between invasive populations from the core and from the front of the invasive area in Western Europe. We were also interested in assessing possible change in this trait in laboratory-reared biocontrol populations of the same species. Finally, we examined whether the norm of reaction of cannibalism to individual density has shifted during the invasion and/or laboratory rearing processes. To address these issues, we collected ladybirds from three types of population: 1) two populations from the native area, 2) six invasive populations sampled along two transects going from the core to the front of the invaded area in Western Europe and 3) two European biocontrol strains. We assessed cannibalism of eggs by unrelated first instar larvae and adult females of *H. axyridis* at two different individual densities, in laboratory-controlled conditions.

## Results

### (a) Spatial expansion in Western Europe

**Figure 1:**
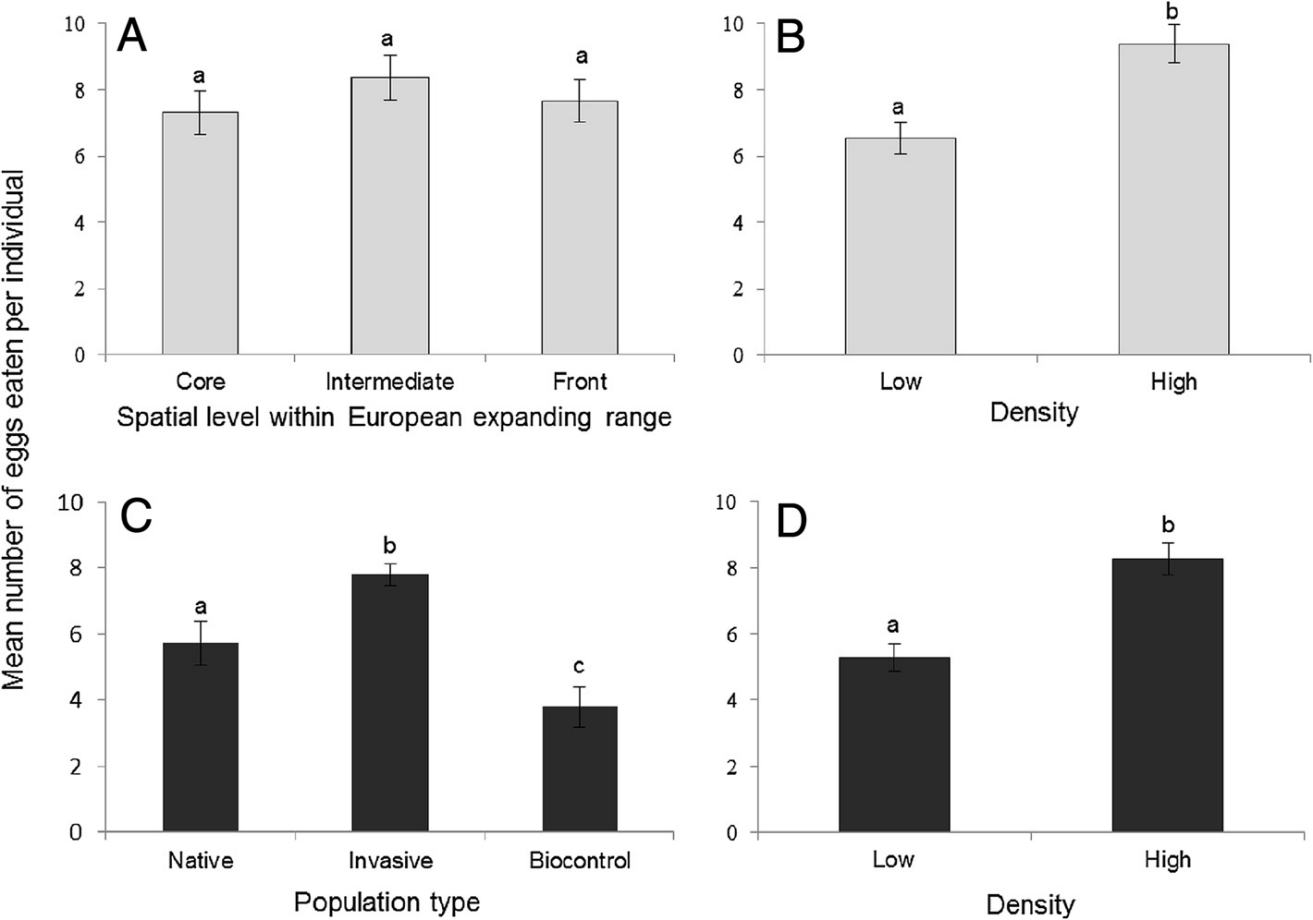
**Mean numbers of eggs eaten per individual (± standard error) by first instar larvae of *H. axyridis*.** Note: Results are given for **(A)** the three spatial levels within Europe expanding range (core, intermediate and front), **(B)** the low and high individual density treatments in data **(A)** pooling geographic locations, **(C)** three types of studied populations (i.e. Native, Invasive and Biocontrol populations), and **(D)** the low and high density treatments in data **(C)** pooling the different population types. Bars marked by different letters are significantly different at the 5% threshold (*P*-levels are mentioned in the main text and Table 1).

**Figure 2:**
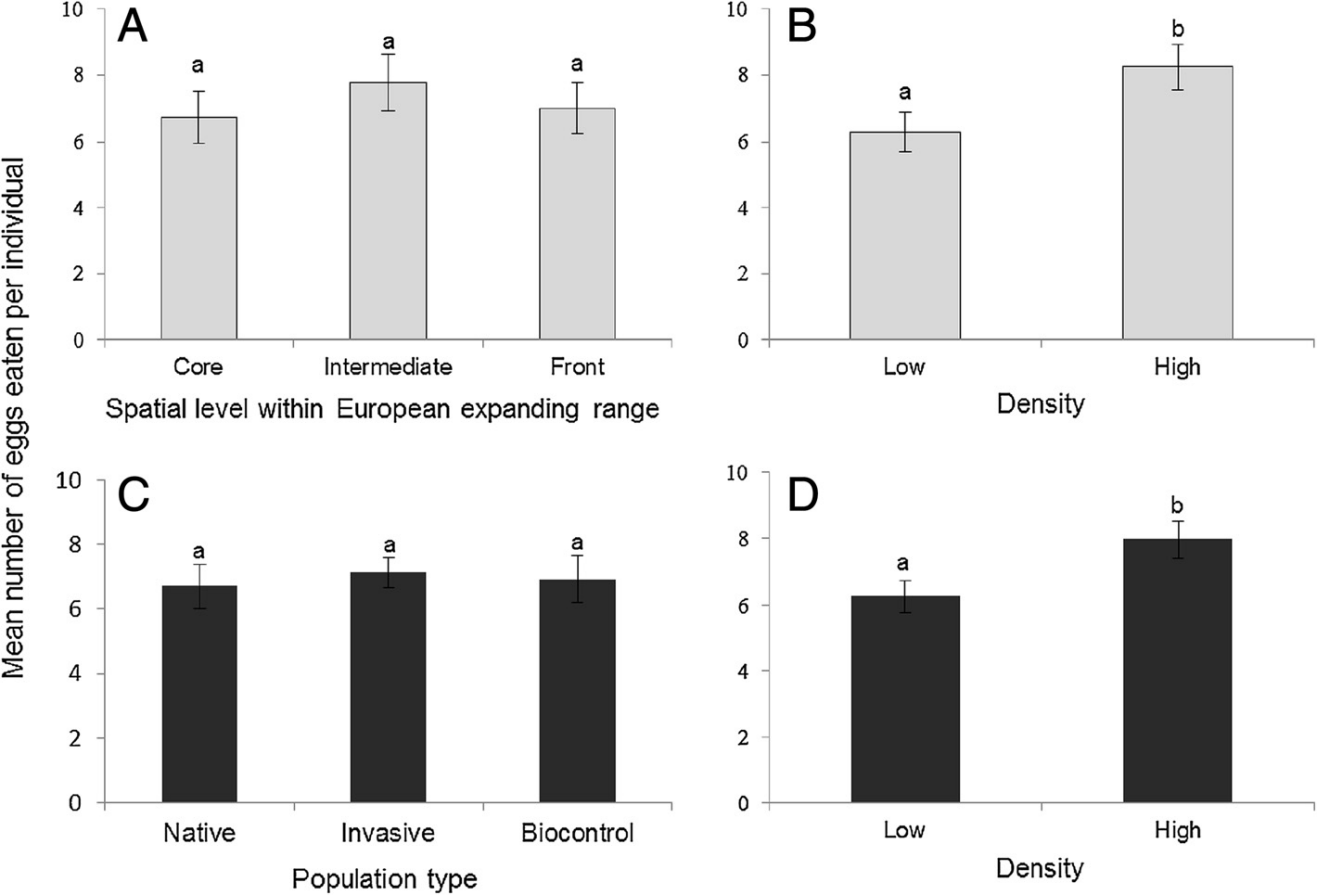
**Mean number of eggs eaten per individual (± standard error) by adult females of *H. axyridis.*** Note: Results are given for **(A)** the three spatial levels within Europe expanding range (core, intermediate and front), **(B)** the low and high individual density treatments in data **(A)** pooling geographic locations, **(C)** three types of studied populations (i.e. Native, Invasive and Biocontrol populations), and **(D)** the low and high density treatments in data **(C)** pooling the different population types. Bars marked by different letters are significantly different at the 5% threshold (*P*-levels are mentioned in the main text and Table 1).

We did not find any significant difference in cannibalism rates by first instar larvae and adult females between populations located on the core, intermediate and front of the expanding range (*P* = 0.52 and *P* = 0.81 for larvae and adults respectively, Table 1 and Figures 1A and 2A). Population identity within Western Europe did not have a significant effect on cannibalism by first instar larvae or adult females (*P* = 0.79 and *P* = 0.93 for larvae and adults respectively, Table 1).

**Table 1:** **Statistical analyses of cannibalism rates by first instar larvae and adult females of *H. axyridis***

	**First instar larvae**		**Adult females**	
**Analysis**	**Test statistic**	**P-value**	**Test statistic**	**P-value**
1- Spatial expansion in Europe	F (df)		F (df)	
Spatial level	0.64 (2)	0.5252	0.20 (2)	0.8173
Population (Spatial level)	0.34 (3)	0.7914	0.15 (3)	0.9279
Density	14.07 (1)	**<0.0002**	1.19 (1)	**<0.042**
Spatial level × Density	2.38 (2)	0.0949	0.78 (2)	0.4556
2- Different types of population				
Population type	17.44 (2)	**<0.0001**	0.19 (2)	0.8262
Population (Population type)	0.59 (7)	0.5106	0.72 (7)	0.6482
Density	22.63 (1)	**<0.0001**	4.34 (1)	**<0.037**
Population type × Density	0.34 (2)	0.7066	0.40 (2)	0.6706

Note: Analysis 1- focuses on spatial expansion in Western Europe (invaded area), and analysis 2-compare different types of populations, i.e. native, invasive and biocontrol. Significant *P*-values at the 5% threshold are shown in bold.

Cannibalism by first instar larvae was globally 43% higher in the high-density treatment compared to the low-density treatment (mean number of eggs consumed per larva in 42 h = 9.39 and 6.55 for high and low density, respectively; *P* = 0.0002, Table 1 and Figure 1B). Cannibalism by adult females was globally 31% higher in the high-density treatment compared to the low-density treatment (8.24 and 6.28 eggs consumed per female per 42 h for high and low density, respectively; *P* = 0.04, Table 1 and Figure 2B). We did not find any significant interaction between the individual density and the core, intermediate and front populations (*P* = 0.09 and *P* = 0.46 for larvae and adults respectively, Table 1). This means that reaction norms according to individual density did not evolve during the spatial expansion, at least in the studied area.

### (b) Native versus invasive versus biocontrol populations

We compared the cannibalism rates among the three types of population (native, invasive and biocontrol). Cannibalism by first instar larvae differed significantly between population types (*P* = 0.0001, Table 1 and Figure 1C). Larvae from invasive populations displayed a 37% higher cannibalism rate compared to those from native populations (7.78 and 5.69 eggs per larva per 42 h for invasive and native larvae, respectively; *P* = 0.002, Figure 1C) and displayed twice the cannibalism rate of larvae from the biocontrol populations (3.79 eggs per larva, *P* = 0.0001, Figure 1C). The native larvae cannibalized 55% more than those from the biocontrol populations (*P* = 0.05, Figure 1C). There was no significant interaction between population types and density level (*P* = 0.71, Table 1). In contrast to the results obtained on first instar larvae, cannibalism by adult females did not differ significantly between population types neither alone (*P* = 0.83, Table 1 and Figure 2C) nor in interaction with density level (*P* = 0.67, Table 1).

Considering the three types of populations together, we found that higher density significantly increased cannibalism by 41% in first instar larvae (mean number of eggs consumed per larva per 42 h = 8.25 and 5.87 for high and low density, respectively; *P* = 0.0001, Figure 1D) and by 27% in adult females (7.96 and 6.25 eggs consumed per female per 42 h for high and low density, respectively; *P* = 0.04, Table 1 and Figure 2D). The factor population identity was again not significant considering the three types of populations (*P* = 0.51 and *P* = 0.65 for larvae and adults, respectively; Table 1).

## Discussion

We provide the first evidence that cannibalism behaviour may differ between native and invasive populations of a given species. More specifically, we found that larvae from invasive populations of *H. axyridis* in Western Europe have a higher propensity for cannibalism than larvae from the native range and from commercial laboratory-reared biocontrol populations. Cannibalism behaviour has been suggested to facilitate the colonization of marginal new environments by rescuing individuals from nutritionally poor situations [11,21]. An evolutionary increase in cannibalism linked to natural selection in such a nutritionally poor environment has been experimentally demonstrated in laboratory experiments [11,22]. A possible hypothesis is thus that upon entry into the new environment of the invasive area, *H. axyridis* larvae may have faced a nutritionally stressful situation (e.g. physiological maladaptation to the new prey, difficulty to find or capture them, or even a lack of temporal synchrony). *H. axyridis* mostly feeds on tree-dwelling hemipteran insects such as aphids, psyllids, and scale insects. It is known that aphid fauna differ between Asia and Europe [35], with however some species which are common between both areas. Plants, that may be used by HA to find its preys, also differ much between the two areas. Identifying the environmental differences that have selected for the observed change in cannibalism propensity would require additional work on HA foraging behavior in both areas.

Although *H. axyridis* is a generalist predator, first instar larvae tend to stay on the leaves on which they hatched [36] and their efficiency in capturing prey is very low even on prey encounter [37,38]. Our results, together with those of Wagner and colleagues [20], who found a higher heritability for larval cannibalism in low food environments as compared to high food environments, support the hypothesis that larval propensity for cannibalism in *H. axyridis* responds to selection in nutritionally poor situations, Another potential explanation for this higher cannibalism rate in invasive larvae could be linked to the presence of defensive alkaloids produced by predatory ladybirds against intra- and interspecific egg predation. Kajita et al. [39] showed that the amount of alkaloids could vary significantly between egg clutches within and among females in *H. axyridis*. It could thus be interesting to test whether the amount of alkaloids in eggs of invasive populations is lower than that in native ones and whether this corresponds with the higher cannibalism rate in invasive populations.

We also found that larvae from laboratory-reared biocontrol populations showed a lower propensity for cannibalism than larvae from wild native or invasive populations. This difference may result from the particular conditions of captive rearing. During mass rearing, biocontrol larvae have been fed *ad libitum* on highly nutritious unnatural prey (*E. kuehniella* eggs) over numerous generations. Such feeding regimes may have suppressed the selective pressure for cannibalism, which therefore substantially declined. It is also possible that cannibalism has been purposefully counter selected during the mass rearing process, with ladybirds displaying the cannibalism phenotype being discarded from the rearing. Although these selective explanations are attractive, we cannot at present exclude the possibility that the observed shift results from genetic drift, which is also common under laboratory rearing conditions [40], particularly as biocontrol populations are often characterized by low effective population sizes. In agreement with this, substantially lower genetic variation was found at microsatellite markers in biocontrol populations when compared to both native and invasive populations [24]. It has to be noted, however, that captive rearing has already been shown to significantly impact several other traits in biocontrol populations of *H. axyridis,* including a higher male reproductive success, a lower survival rate at lower temperatures [41] and a lower resistance to pathogens [42-44]. Altogether, it may indicate that adaptation to laboratory rearing conditions involves important reallocation of resources and energy, which is likely to affect other life history components of *H. axyridis* like fecundity and survival. Interestingly, although Western European invasive populations display a contribution of biocontrol genes estimated around 40% [23,24], these invasive populations showed a significantly higher propensity for cannibalism than biocontrol populations. This result indicates that the low propensity for cannibalism of biocontrol individuals has not been retained in the field but was rather counter-selected during the genetic introgression process.

Our experiments also shed light on the difference in cannibalism evolution with respect to life stages. In contrast to larvae, invasive adults do not show any significant difference in the propensity for cannibalism compared to native and biocontrol ones. In agreement with our laboratory-based results, most of the descriptions of egg cannibalism in the field involve larvae [25,28]. Contrary to young larvae, which often stay on leaves on which they hatched [36], adults are able to fly long distances searching for food and may thus suffer much less from local resource scarcity. Moreover, the energetic advantage of cannibalism could be much greater for larvae than for adults.

A further aim of our study was to test whether the level of cannibalism differs between invasive populations from the core and from the front of the invaded area in Western Europe. A theoretical study has shown that higher dispersal rates can increase the probability of cannibalism but also that cannibalism itself can have important evolutionary consequences and select for increased dispersal rates [8]. Our study did not reveal any significant difference in cannibalism rates from the core to the front of the invasion range in either larvae or adults. For adults, the absence of difference along the expanding range might be explained by the same reasons as those detailed previously. For larvae, however, we suggest that the evolutionary increase of cannibalism between native and Western European invasive populations might have eroded the genetic variance at this trait, preventing a further increase in cannibalism rate along the expanding range. This is even more plausible given that Western European invasive populations correspond to a secondary introduction from a previously invaded area in Western USA [45]. This result indicates that cannibalism has not evolved conjointly with dispersal during the expansion of Western Europe. Rather, as it has been theoretically shown [8], the increased cannibalism rate in the invasive area compared to the native one may have helped the rapid increase in dispersal along the expanding range in Western Europe [8].

Finally, individuals often have to face recurrent bottlenecks (i.e. periods of low population density) during the invasion process, both at the time of the introduction and during the spatial expansion [45]. Because a reduced population density found at an expanding front may potentially reduce the selective advantage of cannibalism, one could have predicted a change of the norm of reaction of cannibalism to individual density during the spatial expansion of an invasive area. For both larvae and adults, we detected a higher individual rate of cannibalism at higher densities. This result is congruent with cannibalism rate being higher at higher densities in the majority of taxa [1,2] and confirms that population density is a key factor in the expression of cannibalism [46]. We did not find however any significant interaction between the density and the tested populations (invasive populations located on the core, intermediate and the front of the expansion). This means that reaction norms according to density did not change during the course of invasion (at the introduction or during the spatial expansion). Interestingly, a similar result was observed for biocontrol populations of *H. axyridis,* which also endured recurrent and severe bottleneck events during their rearing in the lab.

## Conclusions

This study is the first to provide evidence for a higher propensity for cannibalism in natural invasive populations compared to native ones. However, we are still at an early stage in understanding the underlying mechanisms and several research perspectives seem appealing. For instance, since *H. axyridis* now has a worldwide distribution (Asia, Africa, South America, North America, Europe) [47], the study of cannibalism behaviour in invaded continents other than Europe would allow to determine whether the higher propensity for cannibalism in invasive populations is a general feature of the invasion process at the worldwide scale. In addition, it has been suggested that enhanced cannibalism might be a transient phenomenon in the new environment, with cannibalism rates increasing initially and then declining as physiological adaptation to new prey increases and the nutritional benefits of cannibalism thereby diminish [11,22]. It would hence be interesting to test whether higher cannibalism rates are maintained in the European invasive populations in the long term.

## Methods

### (a) Sampling of populations and rearing conditions

**Figure 3:**
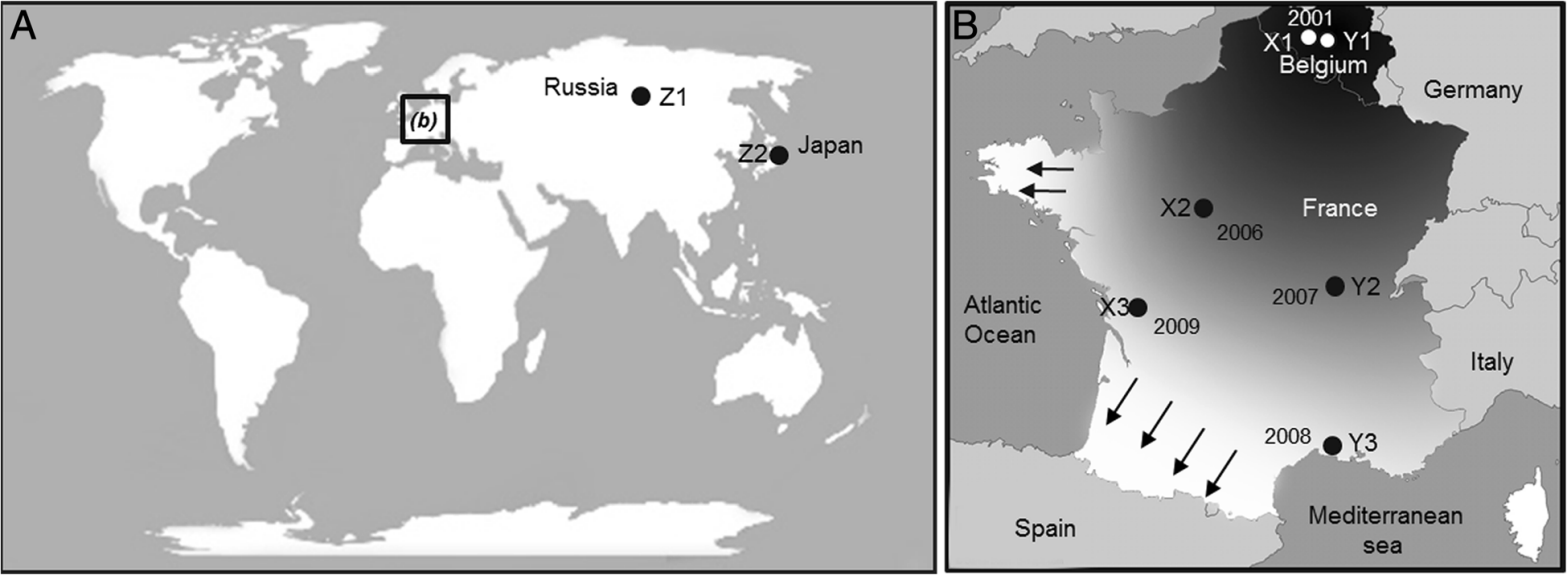
**Geographic locations of the sampled populations of *H. axyridis.*** Note: **(A)** Geographical regions of the different native (Z1 and Z2) and invasive (X1 to X3 and Y1 to Y3) sampled populations of *H. axyridis.* Closed circles correspond to locations where the two native populations have been sampled. **(B)** The Western European invasive populations of *H. axyridis* were sampled along two transects. Letters (X or Y) correspond to the transect names, and years are the dates of the first observation of *H. axyridis* at the sampled geographic localities. Two biocontrol populations reared in European laboratories since 1981, probably originating from China, were also used for the present study. See main text for details.

A total of ten *H. axyridis* populations of three different types (native, invasive and biocontrol) were used in this study. The first observations of established feral *H. axyridis* populations in Europe were made in 2001 near Ghent and Brussels in Belgium. A rapid demographic and spatial expansion to a large part of Europe has subsequently been observed [47,48]. We thus considered the centre of Belgium as the invasion core. Based on the information about the spatial expansion given by the French national *H. axyridis* survey (http://vinc.ternois.pagesperso-orange.fr/cote_nature/Harmonia_axyridis/), we collected six populations, sampled between October and November 2010, along two transects (three samples per transect) ranging from the invasion core of the European outbreak (Brussels area) towards the invasion front in southern and western France (Figure 3, Table 2). The six population samples used in this study are part of the same spatial expansion of a unique outbreak originating from a single introduction event as demonstrated by using analyses based on microsatellite markers. Two samples were also collected from the native range of *H. axyridis* in 2009 (Krasnoyarsk in Russia and Fuchu in Japan). Sampling was conducted in public location that did not require specific authorization, and did not involved endangered or protected species. Finally, we used two *H. axyridis* samples from commercial biocontrol stocks (from Biotop and Biobest biocontrol companies), both of which were derived around 1995 from a strain reared under laboratory conditions since 1982. Founding insects of those laboratory populations were probably collected in China by INRA (Institut National de Recherche Agronomique, France) [24,49]. Before the experiments started, we reared all the populations in the laboratory for at least three generations (G_0_ to G_3_), under strictly controlled conditions, in order to avoid bias due to maternal effects. In all experiments, individuals were fed *ad libitum* with irradiated *Ephestia kuehniella* (Lepidoptera: Pyralidae) eggs and reared at constant environmental conditions (24 ± 1°C; 60 ± 10% relative humidity; L:D 14:10 h photoperiod).

**Table 2:** **Sampling details of the ten studied *H. axyridis *** **populations**

**Biogeographic status**	**Sampling site locality**	**Sample code**	**Coordinates**	**Distance from outbreak core (Km)**	**Date of first observation**
Invasive	Brussels (Belgium)	X1	50.839°N	0	2001
			4.368°E		
	Fondettes (France)	X2	47.402°N	469	2006
			0.637°E		
	Chizé (France)	X3	46.148°N	629	2009
			0.424°W		
	Walhain (Belgium)	Y1	50.612°N	0	2001
			4.668°E		
	Quincieux (France)	Y2	45.909°N	522	2007
			4.758°E		
	Prade-le-lez (France)	Y3	43.698°N	770	2008
			3.863°E		
Native	Krasnoyarsk (Russia)	Z1	56.001°N	N/A	N/A
			92.885°E		
	Fuchu (Japan)	Z2	34.57°N	N/A	N/A
			133.24°E		
Biocontrol	Valbonne (France)	W1	N/A	N/A	N/A
	Westerlo (Belgium)	W2	N/A	N/A	N/A

Note: Two populations were sampled in the native area (Z1 and Z2), six in the European invaded area (X1 to X3 and Y1 to Y3) and two populations originated from commercial biocontrol stocks (W1 and W2). For the European invasive populations, samples were collected along two transects starting from the outbreak core and ending near the invasion front (see Figure 3). N/A – not assessed.

### (b) Experimental procedures

For each population, the G_3_ generation consisted of 200 individuals (100 females and 100 males), which were placed randomly into five boxes (9 cm high, 29 cm length and width) with 20 females and 20 males per box, for ten days to allow for mating. The 200 individuals from these 5 boxes were then distributed between two groups. The first group (60 females and 60 males) was used to produce eggs for the cannibalism experiments. The *H. axyridis* eggs used in these experiments were collected daily and kept at −20°C for a maximum period of two weeks before use in the cannibalism experiments. We collected 2400 eggs for each population sample (totalling 24000 stored eggs). Individuals of the second group (40 females and 40 males for each population sample) were divided over two boxes and were used to obtain larvae and new adults for the cannibalism tests. To do so, we collected 25–35 egg clutches per population sample. A subsample of the larvae obtained from those eggs was used to test the propensity for larval cannibalism on eggs. The remaining larvae were reared to adulthood in order to test the propensity for cannibalism of adult females.

To measure cannibalism rates, we placed individuals in a small cylindrical box measuring 5 cm diameter and 2.5 cm high. First instar larvae and adult females of each population sample were separated into two groups: low density (one individual per box), and high density (five larvae or three adult females per box). Larvae and adults had never consumed Harmonia eggs prior to being placed in the test arena. In the centre of each box we placed 15*H. axyridis* eggs per individual on a piece of black paper (i.e. same ratio of eggs to predator in every box). Note that the eggs and the cannibalistic larvae or adults belong to the same population. We recorded the number of eggs eaten after 42 h. (see Additional file 1: Figure S1 for illustration). The duration of 42 hours relied on practical grounds. We needed this period to allow discriminating between the tested populations. Too short a duration may hamper the behavioral differences to express while too long a duration would result in all eggs being eaten in all treatments. Preliminary experiments enabled us to find the most adequate duration.

A total of 950 first instar larvae (24 hours old) were tested with, for each population, n = 75 replicated tests for the high-density treatment and n = 20 tests for the low-density treatment. A total of 650 adult females were tested with, for each population, n = 45 replicated tests for the high-density treatment and n = 20 test for the low-density treatment. All adult females were 10–15 days old and initially fed with *Ephestia* eggs to standardize their feeding response.

### (c) Statistical analysis

All statistical analyses were conducted using the JMP Pro 9 package (SAS Institute 2009). To study the propensity for cannibalism, the number of eggs eaten by individuals for each of the different treatments was compared using ANOVA, as follows. First, we focused on the six (invasive) European populations to test for potential differences in cannibalism rates that might take place during the spatial expansion in Western Europe. The model included the following factors: spatial level (core, intermediate and front, Figure 3), population sample nested in spatial level, density (low or high) as well as the interaction between density and spatial level. Second, we tested for differences in cannibalism rates among the three different types of *H. axyridis* populations. The model included the following factors: population type (native, invasive and biocontrol), population sample nested in population type, density (low or high) and the interaction between density and population type. Note that the analyses presented here include the six invasive Western European populations, but we obtained the same results when taking two of those populations randomly (results not shown). We performed all analyses separately for first instar larvae and adult females. Data sets for adult females and first instar larvae are provided in additional files 2 and 3, respectively. 

## Competing interests

The authors declare that they have no competing interests.

## Authors’ contributions

All authors read and approved the final manuscript, BF, AT and AE designed the experimental, AT, EL and NK collected samples, AT and BE analysed the data, BF, AT and AE drafted the manuscript.

## Supplementary Material

Additional file 1 **Photos F1 illustrating the cannibalistic behaviour on eggs by (A) a larva (stage L1) and (B) an adult individual of *Harmonia axyridis*.** (Photo courtesy of A. Tayeh). Click here for file

Additional file 2 The data set of cannibalism by adult females. Click here for file

Additional file 3 The data set of cannibalism by first instar larvae. Click here for file
