# Supplementary figures and images for: Cannibalism in invasive, native and biocontrol populations of the harlequin ladybird
Source: BMC Evol Biol. 2014 Feb 5;14:15. doi: 10.1186/1471-2148-14-15 (PMC3913791; doi:10.1186/1471-2148-14-15)

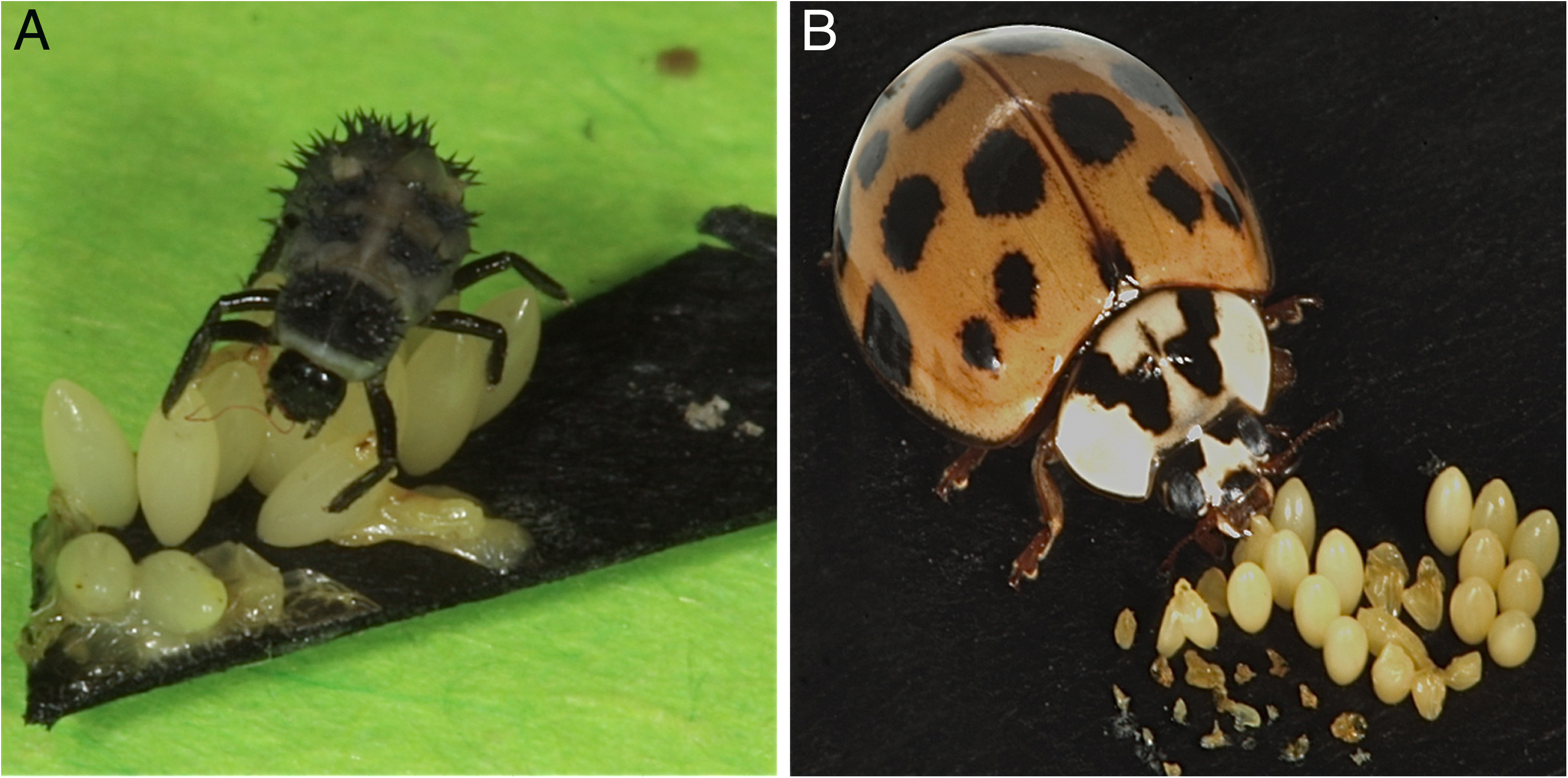

Supplement: Additional file 1 — Photos F1 illustrating the cannibalistic behaviour on eggs by (A) a larva (stage L1) and (B) an adult individual of Harmonia axyridis . (Photo courtesy of A. Tayeh). [file 1471-2148-14-15-S1.tiff]
